# Extended Spectrum Beta-Lactamase Carriage State among Elderly Nursing Home Residents in Beirut

**DOI:** 10.1155/2015/987580

**Published:** 2015-03-18

**Authors:** M. A. Jallad, R. Naoufal, J. Irani, E. Azar

**Affiliations:** ^1^Faculty of Medicine, American University of Beirut, Beirut, Lebanon; ^2^Faculty of Medicine and Medical Sciences, University of Balamand, Beirut, Lebanon

## Abstract

*Introduction.* ESBL-producing Enterobacteriaceae can cause severe infections, but they are also isolated from the stool of asymptomatic subjects. Faecal carriage of such organism is poorly understood.* Methods.* First phase of the study was cross-sectional with prevalence and epidemiology of ESBL faecal carriage in two nursing homes in Beirut: 57 residents in the first (NH1) and 151 residents in the second (NH2). In second phase, faecal swabs from cohort of NH1 residents were examined for carriage at six-week intervals over three-month period. Residents' charts were reviewed to assess carriage risk factors.* Results.* Over 3 consecutive samplings at NH1, 81% of residents were at least one-time carriers with 50% at the first round, 60.4% at the second, and 74.5% at the last one. At NH2, 68.2% of residents were carriers. Constipation (in NH1) and antibiotic intake (in NH2) were significantly associated with higher ESBL faecal carriage while the length of stay at the nursing home (in NH2) was associated with less carriage.* Conclusion.* Faecal carriage of ESBL-producing Enterobacteriaceae is high among nursing home patients in Beirut. The rate of carriage changes rapidly and significantly over time either with multiple factors playing a possible role like outbreak spreading, antibiotic, and health care system exposure.

## 1. Introduction

Beta-lactam antibiotics are extensively used in the treatment of several types of infections [1–3]. Extended spectrum beta-lactamases (ESBLs) are enzymes produced by bacteria and capable of hydrolysing beta-lactam antibiotics [4, 5]. The first ESBLs were identified in the 1980s. Point mutations at the level of the beta-lactamase active site transformed parent, non-ESBL TEM-1, TEM-2, and SHV-1 into ESBL TEM and SHV. Throughout the 1980s and 1990s, the main types of ESBLs were TEM and SHV and were mostly produced by* Klebsiella*. They were mainly isolated in hospital settings from nosocomial infections. However, from the year 2000, a whole shift in the epidemiology took place [6]. ESBLs were also produced by* E. coli* strains and were increasingly isolated in community settings. The main ESBLs became CTX-M type [6]. This new type of ESBL is thought to originate from a bacterial species in the environment:* Kluyvera* [6, 7]. While most ESBL-producing strains were clonally related in the past, the newer CTX-M-type ESBLs producing bacteria were not [8]. ESBL-producing strains are being increasingly isolated throughout time. This can be explained by the easy transmission of CTX-M encoding plasmids through conjugation [9].

In Lebanon, the genes bla_CTX-M_, bla_TEM_, and bla_SHV_ were all found in clinical isolates of ESBL-producing* E. coli* and* Klebsiella *spp. In addition, a 90 kb plasmid was identified [10]. This plasmid can transmit ESBL resistance as well as resistance to quinolones across bacteria since it harbours genes encoding CTX-M15 and AAC(6′)-Ib-cr, which is a quinolone-modifying enzyme [10].

Studies done in Lebanese hospitals showed a constant rise in prevalence of ESBL-producing strains [11]. Statistics from the Saint Georges Hospital University Medical Centre in Beirut showed that, in 1998, 3.3% of* E. coli* and 6.4% of* Klebsiella* strains were ESBL producers (unpublished data). While in 2011, 34% of* E. coli* and 72% of* Klebsiella* strains (isolated from urine) were ESBL producers.

With such high prevalence, ESBL-producing bacteria became the subject of concern of many researchers, owing to two major points. The first point is the hurdle of treatment of infections caused by these pathogens with powerful antibiotics which often necessitates hospitalisation. The second point is the frequent transferability of this resistance among bacteria resulting in increasing rates of ESBL infections and carriage. The means of acquiring ESBL are not yet completely understood with several gaps remaining as to the spread of this resistance in the community and among health care facilities residents. Moreover, ESBL strains are being isolated in nursing homes and other health care associated facilities in the community [12–18]. This suggests the presence of a reservoir for these resistant strains outside hospital wards.

This study seeks to estimate the prevalence of ESBL faecal carriage among nursing home residents in Beirut and how this prevalence varies with time. In addition, this study is an attempt to give insight into the characteristics of ESBL faecal carriers and the risk factors associated with this carriage among nursing home residents.

## 2. Materials and Methods

### 2.1. Study Design

We conducted a cross-sectional study at two different nursing homes in Beirut. In one of the nursing homes, we followed a cohort of patients prospectively in order to determine the change in carriage status and the development of UTI.

### 2.2. Population

The data was collected at the Saint Georges Hospital Nursing Home (NH1) and the Dar Al-Ajaza Al-Islamia Nursing Home (NH2). NH1 included 30 rooms and had a capacity of 70 beds. NH2 had an 800-bed capacity including 400 beds for psychiatry and mental retardation and 400 beds for the elderly. All the individuals who were nursing home residents and who were above the age of 65 were eligible for the study. Individuals who (or whose surrogate) refused to sign the consent form were excluded. Candidates who met the inclusion criteria were systematically included. All residents of NH1 were eligible and signed the consent form. Rectal swabbing was repeated three times among these residents: the first was in April 2012 (*T*_0_); the second was in May 2012 (*T*_1_), and the third was in July 2012 (*T*_2_).

### 2.3. Procedure

The faecal carriage was determined by performing rectal swabbing on all participants. Rectal swabbing was performed since the sensitivity and specificity of this technique were proved to be to a great extent higher than those of stool samples in determining faecal carriage [15]. The swabs were then cultured on specific chromogenic agar media (ChromID ESBL, Biomerieux, Marcy l'Etoile, France). ESBL detection was done according to CLSI guidelines using disk diffusion test for cefotaxime and ceftazidime along with amoxicillin-clavulanic acid [19] for detection of synergy (keyhole effect) in a double disk synergy method. Possible Amp C resistance was detected by cefoxitin disks. Ciprofloxacin disks were used to test for quinolone resistance. The identification of all the strains was done using API 20E kit (Biomerieux, France). Finally, all isolated strains were preserved in a <–80°C freezer in cupules containing a combination of nutrient broth (85%) and glycerol (15%).

The medical records of all participants were checked at the beginning of the study. A questionnaire was used to gather details about patients' age, gender, length of stay (LOS) at the nursing home, diagnosis on admission, mobility status, comorbidities (neurologic diseases, diabetes, cancer, cardiovascular diseases, pulmonary diseases, and underlying urogenital pathologies), bladder/bowel incontinence, indwelling medical devices (permanent and intermittent urinary catheters), and hospital admissions during the last year and antibiotic treatments in the preceding 3 months. At NH1, the faecal carriage was reexamined and the medical records were reviewed every 6 weeks to note any alteration in the medical condition. A case of ESBL faecal carriage was defined as an individual who had at least one positive carriage. All NH1 participants were monitored for the onset of any UTI to examine whether it was an infection with an ESBL-producing strain. A UTI is an infection involving any part of the urinary system, including urethra, bladder, ureters, prostate, and kidneys. A case definition of a UTI consists of clinical (dysuria, urgency, frequency, suprapubic tenderness, and flank pain) and microbiological (pure culture of a single microorganism and a colony count higher than 10^5^ colony forming units per mL) findings.

### 2.4. Statistics and Data Analysis

Classical descriptive methods were used for univariate analysis for the whole sample and according to the study site. Continuous variables (age, length of stay) were described using means, medians, and standard deviations. Categorical data were summarized using proportions. Additionally, bivariate analysis was performed to compare different variables according to the carriage status. Quantitative data were compared using Student's *t*-test or Mann-Whitney *U* test when appropriate. Qualitative data were compared using Chi-square or Fisher's exact test when appropriate. Analysis was performed for each site alone; *P* values below 0.05 were considered statistically significant. No adjustment for multiple comparisons was made. All statistical calculations were performed using SPSS V20 (IBM SPSS Statistics 20.0).

## 3. Results

### 3.1. Prevalence and Dynamics of ESBL Faecal Carriage

The overall number of individuals included in the study at least once during the 3 phases at NH1 was 57. At NH2, a one-time rectal swabbing took place in August 2012 and 151 residents (75.5% of eligible individuals) signed the consent form. The participants' demographic and medical data are presented in Table 1. In NH1 at *T*_0_, 26 of 52 residents (50%) were positive for ESBL faecal carriage. At *T*_1_, 32 of 53 residents (60.4%) were positive. Finally at *T*_2_, 38 of 51 residents (74.5%) were positive. Overall, 46 of 57 individuals (80.7%) were at least one-time carriers during the follow-up time, while only 11 of 57 individuals (19.3%) were never carriers. Between *T*_0_ and *T*_1_, the ESBL faecal carriage disappeared in 5 residents (10.2%) while 9 residents (18.4%) acquired this carriage. Between *T*_1_ and *T*_2_, the carriage disappeared in 3 residents (6%) while it was acquired by 10 residents (20%). At NH2, the number of ESBL faecal carriers was 103 of 151 participants (68.2%). In NH1 and NH2 combined, 149 of 208 participants (71.6%) were carriers.

**Figure 1:**
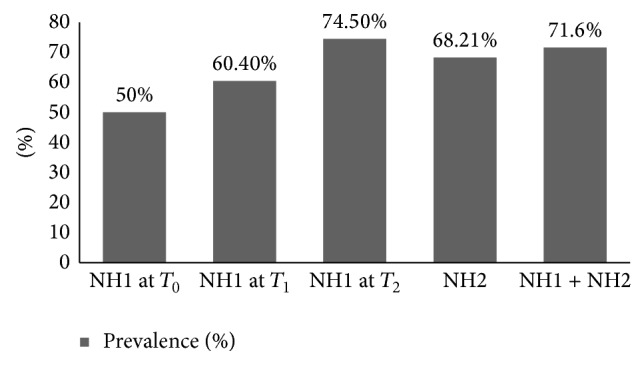
Percentages of prevalence of ESBL faecal carriage.

**Figure 2:**
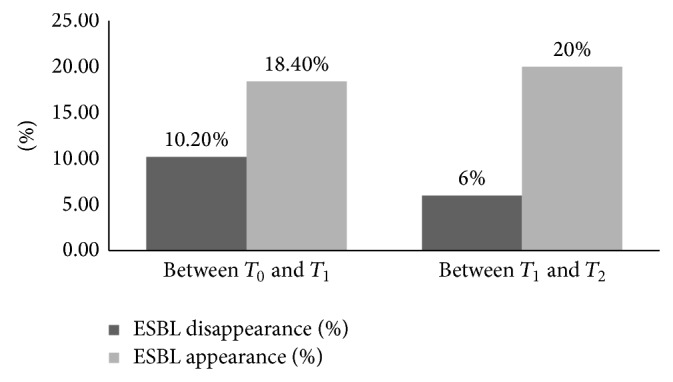
Dynamics of the ESBL faecal carriage at NH1.

Figure 1 summarises the prevalence of the carriage and Figure 2 shows its dynamics between the different sampling times.

### 3.2. Characterisation of the Isolated Strains

Some positive carriers had more than one bacterial strain producing ESBL (up to 4 strains). In total, 226 strains were isolated and preserved and were distributed as follows: 187* E. coli* (82.7%), 22* Klebsiella* (9.7%), 9* Enterobacter* (4%), 4* Citrobacter* (1.8%), 3* Proteus* (1.3%), and 1* Serratia* (0.5%). On average, out of 199 positive samples, 75 samples had resistance to ciprofloxacin (38%) and 29 samples had at least one strain resistant to cefoxitin (15%) hence a possible AmpC resistance.

### 3.3. Incidence of UTI

Throughout the period of the 3 consecutive samplings at NH1, 9 individuals developed a UTI (15.8%), 5 of which were caused by non-ESBL-producing strain (8.8%) while 4 were caused by ESBL-producing strain (7%).

### 3.4. Risk Factors for ESBL Faecal Carriage

The association between different factors and the ESBL faecal carriage at NH1 and NH2 is presented in Table 2. As for NH1, a statistically significant association was noted between constipation and ESBL faecal carriage (*P* value of 0.049). The constipated individuals were 100% carriers while the nonconstipated are 74.4% carriers. Moreover, the chart review of the NH1 residents showed that 15 of the 51 residents (29%) used quinolones in the 3 months prior to the first sampling; 5 out of the 53 residents (9.4%) were exposed between samples one and two, but only 3 out of the 51 subjects (5.8%) were prescribed quinolones between the second and third sampling. At NH2, statistical significance was found for the association between LOS and ESBL faecal carriage with a *P* value of 0.007. Also at NH2, statistical significance was shown for the association between any antibiotic intakes during the last three months before the study with a *P* value of 0.036. Residents who have an antibiotic intake are 87% carriers and those with no antibiotic intake are 64.8% carriers.

## 4. Discussion

There has been little published data from Lebanon looking into the prevalence of ESBL faecal carriage; a study by Moubareck et al. [20] inspected the carriage in ICU patients from 5 hospitals, 58 healthcare workers and 382 healthy subjects, in the year 2003. The prevalence showed 16% carriage in ICU patients, 3.4% in health care workers, and 2.4% in healthy subjects.

Prevalence in long-term care facilities and NHs, in nonoutbreak settings, varied significantly from 1.9% in France [17/877, 2012] [21] to 64% in Italy [71/111, 2009] [15] and 40.5% in UK [119/294, 2009] [14]. The particularly low prevalence in France can be attributed to poor screening method as stated in the study itself. Another study in France [22] showed that prevalence in asymptomatic young adults grew from 0% in 1999 to 2.1% in 2009. The prevalence of ESBL faecal carriage among healthy individuals fluctuated between countries: in Egypt 63% [400/632, 2011] [23], in Spain 6.7% [7/105, 2009] [18], in Saudi Arabia 12.3% [62/505, 2009] [17], in China 7% of elderly people [19/270, 2008] [16], and in Thailand 51.8% [73/141, 2010] [24]. The carriage rate was affected by exposure to health care system. In the Republic of Korea, for instance, prevalence in elderly healthy individuals was 23% [9/39, 2012] while prevalence in hospitalised individuals of the same age group was 49.1% [28/57, 2012] [25]. In Cameroon as well, prevalence in student volunteers was 6.7% [10/150, 2012] while prevalence in outpatients was 23.1% [48/208, 2012] [26].

In our study a high incidence of detection ESBL-producing Enterobacteriaceae was detected at both nursing homes. At NH1 the prevalence was 50% with 73% of the isolates ciprofloxacin resistant at the first sampling. The prevalence increased to 60.4% six weeks later, but ciprofloxacin resistance decreased to 44%. On our last sampling, the proportion of residents carrying ESBL in their stool increased to 74.5%, but a minority of 27% continued to be ciprofloxacin resistant. Assuming that there were no technical and sampling variations, this rise may well be the result of an outbreak with an ESBL-producing but ciprofloxacin sensitive organism at the concerned NH. The consumption of quinolones decreased markedly from 29% in the 3 months prior to the first sampling to 9.4% in the first 6 weeks of study and to 5.8% in the last 6 weeks; this may have affected the profile of resistance to ciprofloxacin in ESBL-producing strains, but the small numbers are not enough to confirm such a conclusion. The distribution of strains found in our study is similar to that in the literature. Studies inspecting rectal colonisation with ESBL-producing strains as well as studies examining the isolation of such strains from clinical samples show a similar strain distribution [11, 15, 17, 19, 23, 27].

During the 3-month follow-up time of the study at NH1, 9 residents (15.8%) developed a urinary tract infection; 4 were due to ESBL-producing strains and 5 due to non-ESBL-producing organisms. All 4 participants who had UTI with ESBL-producing organisms were females; their infection was mild and they did not require hospitalisation for their infection or any other reason during the study time. Two subjects were carriers during the 3 samplings; the other two were carriers before the development of their infection, but that carriage disappeared on further follow-ups. The relation between carriage and infection has been validated in multiple studies [5, 9, 28–30], though our small sample prevents us from firm conclusions in that regard. In a study by Daoud and Afif [31] assessing all UTIs of both inpatients and outpatients at our university medical centre, Saint Georges Hospital, from year 2000 till year 2009, a considerable rise is shown in ESBL prevalence from 2.3% in 2000 to 16.8% in 2009. The proportion of urinary tract infections due to ESBL* E. coli* strains rose exponentially from 2000 till 2006 and then stabilised at 19.2% out of 688 isolates in 2007, 17.1% out of 727 isolates in 2008, and finally 16.8% out of 628 in 2009. The great variability in the faecal carriage of ESBL-producing organisms and their ciprofloxacin resistance profile contrasted with a fairly steady burden of disease due to ESBL-producing organisms in urinary tract infection; in addition the known complexity of the intestinal microbiota may lead us to consider faecal carriage as dynamic with acquisition, loss, and coexistence of multiple strains. Such a conclusion will need to be verified in a large prospective cohort study on populations from different areas in Lebanon over a longer period of time.

**Figure 3:**
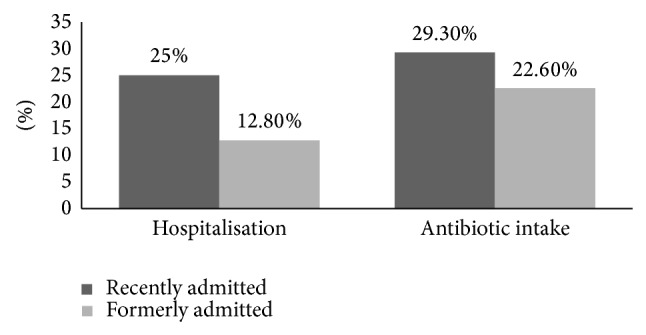
Percentages of hospitalisation and antibiotic intake in recently admitted residents versus formerly admitted residents.

NH1 and NH2 are both situated in Beirut. They are similar in mean age of residents and gender distribution but different in median stay, room accommodations, and hospitalisation scheme and antibiotics intake (Table 1). Therefore the data of each nursing home was analysed separately. Characteristics associated with ESBL faecal carriage in elderly nursing home residents in Beirut (Table 2) were constipation in NH1, antibiotic intake in the last 3 months in NH2, and length of stay in the nursing home in NH2. There is no published correlation between constipation and faecal carriage, but constipation is a known risk factor for development of UTIs that subsequently can affect the faecal carriage. Exposure to any antibiotic in the 3 months prior to the faecal sampling is associated with a higher detection of ESBL-producing organism; such association was demonstrated in many studies [14–16, 21, 32, 33]. Several studies identify quinolone intake, and specifically ciprofloxacin, as a risk factor for the development or persistence of ESBL faecal carriage [12, 26, 34]. Finally a longer length of stay at the nursing home was associated with a lower risk of carriage; comparable findings were previously reported by Rooney et al. [14]. Twenty-five percent of newcomers were recently hospitalised as compared with 12.8% of long-term residents (*P* value = 0.046). A higher percentage of antibiotic exposure was noted in the newcomer residents (29.3%) than in the formerly admitted (22.6%) but does not reach significance (Figure 3). To note that antibiotic consumption was calculated based on patient reporting and nursing home charts this may have underestimated the intravenous antibiotherapy given during hospitalisation. Patients with short nursing home stays may be fundamentally different from patients with long nursing home stays. For example, patients with short nursing home stays reflect a mix of both: patients with recent hospitalizations, surgeries, or medical interventions with high antibiotic use and the intention that most will go home in a short period of time and elderly relatively healthy individuals who will eventually go on to become long-term nursing residents. It is possible that this first group of patients may have transient or short-term ESBL carriage and thus only pose a risk to others in the short term, whereas it is possible that the long-term residents might be chronic carriers and thus pose a greater risk to the community.

In conclusion, stool carriage with ESBL-producing Enterobacteriaceae is high among nursing home residents, playing possibly a role in the endemic status of ESBL urinary tract infection. A higher carriage was noted in newcomers with recent exposure to health care system and antibiotics. These findings are another good reason to conduct larger studies looking into the dynamics and implications of fecal carriage.

## Figures and Tables

**Table 1:** Characteristics of the participants from NH1 and NH2^1^.

	NH1	NH2
Total number	57	151
Gender		
Male	19 (33.3)	54 (35.8)
Female	38 (66.7)	97 (64.2)
Age (years) mean (±SD)	84.8 (±5.3)	77.9 (±7.9)
LOS (months) median (interquartile range)	25 (28.5)	32 (60)
Room accommodation		
Single	13 (22.8)	10 (6.6)
Double	19 (33.3)	4 (2.6)
Triple	9 (15.8)	54 (35.8)
Quadruple	16 (28.1)	1 (0.7)
More than 4 beds/room	None	82 (54.3)
History of UTI (in the prior 12 months)	16 (28.1)	8 (5.3)
Non-ESBL UTI (in the prior 12 months)	14 (24.6)	§
ESBL UTI (in the prior 12 months)	7 (12.3)	§
History of hospitalization during the prior 12 months	28 (49.2)	4 (2.6)
History of antibiotic use during the prior 3 months	29 (50.8)	23 (15.2)
Comorbidities		
Cardiovascular	45 (78.9)	§
Pulmonary	6 (10.5)	§
Diabetes mellitus	12 (21.1)	§
Neurologic	26 (45.6)	§
Renal	7 (12.3)	§
Cancer	3 (5.3)	§
Urogenital pathology	24 (42.1)	§

^1^All the data presented in the table is by number (%) unless stated otherwise.

^§^Data unavailable.

**Table 2:** Association between different factors and the ESBL faecal carriage at NH1, NH2 and both combined^1^.

	NH1		NH2	
	Carriers^*^	Noncarriers	Carriers	Noncarriers
Total number	46 (80.7)	11 (19.3)	103 (68.2)	48 (31.8)
Male	16 (34.8)	3 (27.3)	36 (35)	18 (37.5)
Age (years) mean (±SD)	84.3 (±5.2)	87.4 (±5.2)	78.4 (±7.5)	77.2 (±8.7)
LOS (months) median (interquartile range)	24 (28.8)	25 (32)	32(48)^†b^	50(117)^†b^
Room accommodation				
Single	10 (21.7)	3 (27.3)	8 (7.8)	2 (4.2)
Double	16 (34.8)	3 (27.3)	3 (2.9)	1 (2.1)
Three or more	20 (43.5)	5 (45.4)	92 (89.3)	45 (93.7)
Patient characteristics				
Urinary continent	14 (30.4)	1 (9.1)	27 (26.2)	12 (25)
Bowel continent	16 (34.8)	1 (9.1)	29 (28.2)	13 (27.1)
Suffering from constipation	14(30.4)^†a^	0(0)^†a^	3 (2.9)	1 (2.1)
Bedridden or in wheelchair	11 (23.9)	6 (54.5)	‡	‡
History of UTI				
Absent	22 (47.8)	4 (36.4)	97 (94.2)	46 (95.8)
Less than twice in 6 months	9 (19.6)	3 (27.2)	4 (3.9)	1 (2.1)
More than twice in 6 months	15 (32.6)	4 (36.4)	2 (1.9)	1 (2.1)
Hospitalized during the prior 12 months^‡^	14 (56)	10 (38.5)	4 (3.9)	0
With any antibiotic intake within the prior 3 months^‡^	14 (53.8)	12 (46.1)	20(19.4)^†c^	3(6.3)^†c^

^1^All the data presented in the table is by number (% within dependent variable) unless stated otherwise.

^*^NH1 data is based on the case definition of the faecal carriage that is being at least 1-time carrier through follow-up.

^†^A statistically significant difference (*P* value <0.05) between the ESBL carrier and ESBL noncarrier cases was identified (^a^ *P* = 0.049, ^b^ *P* = 0.007, and ^c^ *P* = 0.036).

^‡^Data unavailable.
